# From Innate to Adaptive: Paradigm Shifts and Frontier Challenges in Next-Generation Vaccine Design

**DOI:** 10.3390/vaccines14030228

**Published:** 2026-02-28

**Authors:** Siqi Huang, Shaochen Yu, Mengjie Zhang, Yuting Huang, Beibei Tian, Jian Lu

**Affiliations:** 1 Department of Oncology, Guilin Hospital of the Second Xiangya Hospital, Central South University, Guilin 541002, China; huangsq23@163.com; 2 Department of Emergency and Critical Care Medicine, Chuzhou Integrated Traditional Chinese and Western Medicine Hospital, No. 788, Huifeng East Road, Nanqiao District, Chuzhou 239000, China; sey_yushaochen@163.com; 3 Department of Gastroenterology, The First Affiliated Hospital of Anhui Medical University, No. 218, Jixi Road, Shushan District, Hefei 230022, China; zmengjie1995@163.com (M.Z.); huangshuishuii@163.com (Y.H.); tianbeibei181@163.com (B.T.)

**Keywords:** innate immune programming, germinal center dynamics, structure-based vaccine design, mRNA delivery platforms, systems vaccinology, broadly neutralizing antibodies, mucosal immunity, immune memory durability, artificial intelligence, nanovaccines

## Abstract

The unprecedented success of mRNA vaccines during the COVID-19 pandemic marks a fundamental paradigm shift in vaccinology, moving the field from empirical pathogen modification toward the rational engineering of host immunity. This review synthesizes recent breakthroughs to construct a conceptual framework for understanding how modern vaccines function as programmable immune instructions. We first analyze the innate immune system as an instructional center, where recognition of vaccine components dictates the quality of ensuing adaptive responses. We then examine the germinal center (GC) as a micro-evolutionary engine for antibody maturation, the output of which can be tuned by vaccine design. The discussion centers on three integrated pillars of next-generation vaccines: computationally designed immunogens, spatiotemporally controlled adjuvant systems, and intelligent delivery platforms, emphasizing that their synergy is essential for achieving broad, durable protection against complex pathogens. Finally, we explore how the convergence of systems vaccinology, artificial intelligence, and personalized medicine is guiding the field toward a more predictable and rapid-response future, while also outlining key advances and persistent challenges.

## 1. Introduction

The evolution of vaccines is, in essence, a grand epic of humanity’s endeavor to understand and harness its own immune defense system. From Edward Jenner’s cowpox inoculation based on folk observation to Louis Pasteur’s rabies vaccine created through directed attenuation, and the monumental successes of traditional vaccines were largely founded on an empirical basis of empirical imitation or controlled simplification of natural infection [1]. This path achieved brilliant successes against pathogens like smallpox, poliovirus, and measles. However, it has repeatedly faltered against pathogens such as *Human Immunodeficiency Virus* (HIV), *influenza virus* with high antigenic drift and shift, *Respiratory Syncytial Virus* (RSV), and *Mycobacterium tuberculosis* with complex intracellular survival strategies [2]. These pathogens evade immunity through high mutation rates, establish latent infections, or possess surface antigens too weakly immunogenic to elicit effective long-term protective memory, profoundly exposing the inherent limitations of development paths based on pathogen culture, empirical inactivation, or attenuation.

The global public health crisis triggered by SARS-CoV-2 and the record-breaking successful deployment of novel vaccine technologies, notably messenger RNA (mRNA) vaccines, dramatically heralded a historic inflection point [3]. This was not a serendipitous technological fortune but the inevitable outcome of convergent scientific progress. The core philosophy of vaccine development has fundamentally shifted from the empirical question—“What immunogen can we derive from the pathogen?”—to an engineering mindset: “How should we design a precise set of biological instructions to programmatically guide the immune system to generate the protective response we anticipate, potentially superior to natural infection in some aspects?” This profound paradigm shift is rooted in decades of convergent breakthroughs and deep integration across immunology, structural biology, genomics, bioinformatics, bioengineering, and, most recently, the success of mRNA–lipid nanoparticle platforms which epitomize the translation of basic science into deployable technology.

We now understand that the innate immune system is far from a simple, non-specific danger alarm. It is a sophisticated biological information-processing center capable of complex decoding, integration, and generation of specific instructions based on the invader’s molecular signature [4]. Antigen-presenting cells (APCs), particularly sentinel dendritic cells (DCs), directly write the fate script for naïve T cell differentiation through the combination, intensity, and spatiotemporal sequence of signals perceived by their array of pattern recognition receptors (PRRs). This determines the global characteristics, strength, and quality of subsequent humoral and cellular immunity. Concurrently, in-depth analysis of the germinal center (GC)—the evolutionary microprocessor of adaptive immunity—has revealed the quantitative rules governing intense clonal competition, somatic hypermutation of antibody genes, affinity-based positive selection, and final fate decisions of B cells [5]. The quality and output of this process are strictly dependent on the physicochemical properties, spatial conformation, valency, and persistence time of vaccine antigens in lymphoid tissues.

This means that every component of a modern vaccine—the precise molecular conformation and 3D spatial arrangement of the antigen, the chemical nature of the adjuvant and its specific intracellular signaling pathways, the physical dimensions/surface charge/biodegradability of the delivery system and its intelligent ability to target specific cells or tissues—has become a tunable parameter for precisely programming complex immune response networks [6,7]. Therefore, next-generation vaccine development is a quintessential multi-objective optimization systems engineering task. It requires the synergistic pursuit of breadth (covering evolving antigenic variants), depth (inducing potentially lifelong immune memory), precision (establishing efficient defense at specific tissue sites like the respiratory mucosa), and agility (rapid response to emerging and re-emerging infectious disease threats). This necessitates conceptualizing and constructing antigen, adjuvant, and delivery system not as independent functional units, but as a functionally deeply coupled, synthetic immune instruction unit requiring global optimization in design.

Following the logical thread of “mechanistic insight driving design innovation, integrated solutions addressing core challenges, and frontier technologies outlining prospects”, this review provides an in-depth synthesis and interpretation of this revolutionary and exciting frontier in vaccinology. We aim not only to present a panoramic view of technological development but also to delve into the deep logical chains connecting basic scientific discovery to cutting-edge application, analyze the immunological principles behind current successes, and critically examine fundamental bottlenecks. We intend to provide readers—whether immunology researchers, vaccine developers, or public health policymakers—with a comprehensive reference combining theoretical depth, technical detail, and strategic vision, to collectively consider how to translate human understanding of the immune system into more powerful tools for safeguarding global health.

## 2. Decoding Protective Immunity: From Linear Chains to Dynamic Network Models

Rational design and precise modulation of vaccines require deconstructing and reconstructing the complex post-vaccination immune response from a “black box” or simple linear causality (vaccination → antigen presentation → lymphocyte activation → antibody production) into a multi-layered, multi-nodal dynamic and tunable network model incorporating positive/negative feedback, nodal synergy, and time-dependent interactions. Fine-grained analysis and quantitative understanding of this network form the biological foundation for all subsequent engineering intervention strategies.

### 2.1. Innate Immunity: Signal Decoding, Integration, and Metabolic Reprogramming as an Instructional Center

The immunological prologue of vaccination begins with the innate immune system’s molecular identity recognition of the vaccine formulation. The antigenic protein/nucleic acid, deliberately added adjuvant molecules, and the physicochemical properties of the delivery vehicle (e.g., lipid nanoparticles, virus-like particles) collectively constitute a unique set of “damage-associated molecular pattern” (DAMPs) or “pathogen-associated molecular patterns” (PAMPs). This complex molecular code is specifically recognized and decoded by professional APCs, particularly various DC subsets strategically distributed in skin, mucosal barriers, and lymphoid organs, via their extensive family of PRRs located on the cell surface, endosomal membranes, and cytoplasm (e.g., Toll-like receptors TLRs, C-type lectin receptors CLRs, RIG-I-like receptors RLRs, NOD-like receptors NLRs, and cytosolic DNA sensors like cGAS-STING) [8,9,10].

**Figure 1:**
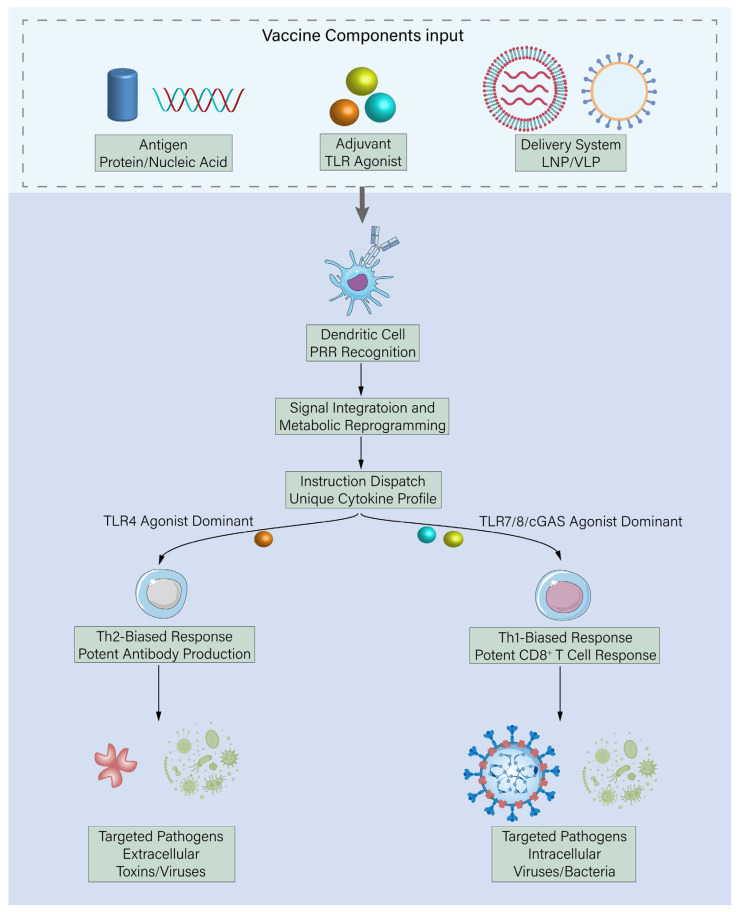
The innate immune system as an instructional centre: Signal decoding of vaccine components and programming of adaptive immunity. Vaccines are complex formulations whose components (Antigen, Adjuvant, and Delivery System) collectively present a unique set of molecular patterns. Dendritic Cells (DCs), the sentinel APCs, decode this information through their repertoire of Pattern Recognition Receptors (PRRs). The specific combination, timing, and intracellular location of these signals trigger distinct downstream pathways and metabolic reprogramming within the DC. This leads to the secretion of a defined cytokine and chemokine profile. For instance, a signal dominated by TLR4 agonists (e.g., MPL) promotes a Th2-biased response, ideal for combating extracellular pathogens. Conversely, signals from endosomal TLR7/8 or cytosolic sensors (e.g., cGAS) drive a Th1-biased response, characterized by strong cell-mediated immunity, crucial for eliminating intracellular infections. Thus, the innate immune system does not merely sound an alarm; it actively programs the quality (Th1/Th2 bias) and magnitude of the subsequent adaptive immune response based on the vaccine’s molecular signature.

The core biological significance of this recognition lies in the extreme specificity and spatiotemporal combinatorial nature of the signaling pathways. Different PRR agonists activate distinct downstream signal transduction networks, leading to profound metabolic reprogramming of DCs, secretion of unique cytokine/chemokine profiles, and upregulation of specific co-stimulatory molecules (e.g., CD40, CD80, CD86). For instance, TLR4 agonists (e.g., monophosphoryl lipid A, MPL) tend to drive strong antibody responses and Th2-biased helper T cell differentiation, suitable for pathogens requiring potent humoral immunity to neutralize extracellular toxins or viruses [11]. In contrast, TLR7/8 agonists (e.g., Resiquimod) or agonists of cytosolic DNA sensors like cGAS (e.g., cyclic dinucleotides, CDNs) potently induce type I interferons (IFN-α/β) and interleukin-12 (IL-12), thereby preferentially promoting Th1-type responses and the generation, expansion, and functional activation of cytotoxic CD8+ T cells, which are crucial for clearing intracellular viruses, bacteria, or protozoa [12]. Furthermore, the timing and subcellular localization of signals are critical: certain adjuvants administered before antigen (priming) or co-administered but released sequentially in different intracellular compartments (e.g., endosome vs. cytosol) of the same APC can produce markedly different or superior immune programming effects. Thus, modern adjuvant science has evolved from pursuing non-specific “potentiation” to aiming for precise “spatiotemporal programming” [13]—actively shaping and “customizing” the “quality” (Th1/Th2/Th17/Tfh bias) and “quantity” (magnitude and breadth) of the adaptive response via rational design of specific signal molecule combinations, precise dose ratios, and controlled release kinetics to optimally match the pathogen’s clearance mechanism (Figure 1).

### 2.2. GC: The Darwinian Evolutionary Crucible for Antibody Quality and Memory Formation and Its Regulatory Hubs

DCs, educated, activated, and metabolically altered by innate immunity, migrate to the T cell zone of draining lymph nodes to present processed antigen peptide-MHC complexes to naïve CD4+ and CD8+ T cells, igniting the adaptive immune engine. The GC reaction is the absolute core for generating high-quality, durable humoral immunity; its operating mechanism is akin to “Darwinian evolution” on a microscopic spatial and temporal scale. Antigen-activated B cells, with initial help from follicular helper T (Tfh) cells, enter primary follicles and proliferate to form GCs. In the GC dark zone, these B cells undergo rapid somatic hypermutation, randomly altering their B cell receptor (BCR) variable region gene sequences to generate a highly diverse B cell clone repertoire [14]. These B cells with mutated BCRs then migrate to the light zone, where they face dual stringent selection pressures: first, they must compete with their nascent BCRs to bind native conformational antigen retained long-term as immune complexes on the surface of follicular dendritic cells (FDCs); second, they must simultaneously receive vital survival, proliferation, and differentiation signals from cognate Tfh cells via surface molecules (e.g., CD40L, ICOS ligand) and secreted cytokines (notably IL-21) [15,16].

**Figure 2:**
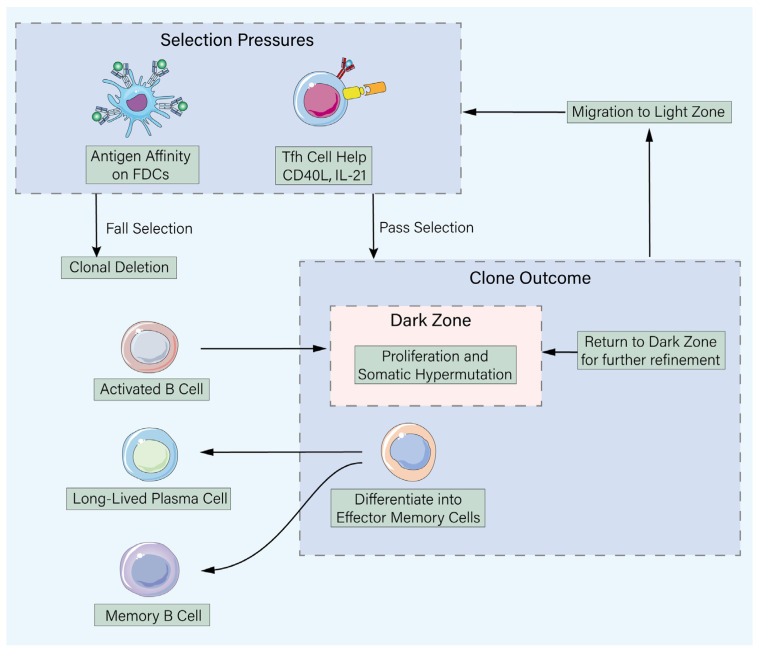
The germinal center reaction: A Darwinian micro-evolution engine for antibody maturation. The germinal center (GC) is a specialized microstructure within lymphoid tissues where B cells undergo a Darwinian process of mutation and selection to produce high-affinity antibodies and memory. 1. Expansion & Diversification: Antigen-activated B cells enter the GC dark zone, proliferate rapidly (forming centroblasts), and undergo somatic hypermutation to randomly diversify their B cell receptor genes. 2. Selection: B cells then migrate to the light zone, where they face dual selection pressures: their mutated BCRs must compete for binding to native antigen displayed on follicular dendritic cells, and they must receive essential survival signals from cognate follicular helper T cells. 3. Outcomes: Clones that fail these criteria undergo apoptosis. Successful “winner” clones have two fates: they can differentiate into long-lived plasma cells (secreting high-affinity antibodies) or memory B cells, or they can re-enter the dark zone for further rounds of mutation and selection, thereby progressively refining antibody affinity. Vaccine design strategies (e.g., nanoparticle display, sustained release) aim to optimize the duration and quality of this GC reaction to elicit superior and broader protection.

Only those B cell clones that, through random mutation, serendipitously acquire higher antigen-affinity BCRs and thereby can more effectively present antigen to Tfh cells to secure sufficient help signals are positively selected, escaping apoptosis [17]. These winners can then re-enter the dark zone for further rounds of mutation and selection (proliferation-mutation-selection cycles), or ultimately differentiate into two key immune memory products: long-lived plasma cells (primarily migrating to bone marrow niches, where they secrete high-affinity antibodies for years to decades, maintaining serum antibody levels) and memory B cells (in a quiescent state but with the potential for rapid reactivation, proliferation, and differentiation into plasma cells upon re-exposure). The excellence of vaccine design is directly and profoundly reflected in its ability to optimize the efficiency, speed, and output quality of this evolutionary process. For example, engineering antigens into multivalent, spatially ordered nanoparticles (e.g., virus-like particles based on ferritin or IMX313 scaffolds) not only provides superior initial B cell activation signals via potent BCR cross-linking but also, due to their nano-size (20–100 nm) and rigid structure, facilitates active lymphatic drainage and prolonged retention on FDC surfaces, providing a stable antigen depot for the weeks-long GC selection process [18,19]. More importantly, prolonging antigen exposure in lymphoid tissues via advanced sustained-release delivery technologies (e.g., biodegradable PLGA microspheres, thermosensitive hydrogels) [20] effectively extends the GC training and selection period, allowing more rounds of B cell mutation and screening, thereby significantly increasing the probability of selecting rare B cell clones capable of recognizing cryptic, highly conserved epitopes (key for future viral variants) or possessing superior neutralizing breadth and potency [21] (Figure 2).

### 2.3. Tissue-Resident Memory: Strategic Significance of Local Barrier Defense and the Resurgence of Mucosal Vaccinology

Although high serum antibody titers and circulating memory lymphocytes constitute the backbone of systemic defense, establishing a first line of rapid, efficient physical-immune barrier at the portal of entry holds irreplaceable strategic value for the vast majority of pathogens invading via respiratory, gastrointestinal, or urogenital mucosal surfaces. Tissue-resident memory T cells (Trm) are the elite guardians of this front-line [22,23]. They no longer circulate between blood and secondary lymphoid organs but, via expression of specific homing and retention molecules (e.g., CD69, CD103), reside permanently or long-term in local tissues (e.g., lung epithelium, intestinal lamina propria, vaginal mucosa). They can be rapidly activated by local antigen very early upon pathogen invasion, proliferate in situ, and execute potent effector functions, potentially even achieving sterilizing immunity by completely blocking infection establishment before any symptoms or transmissibility emerge.

However, traditional systemic vaccination routes (e.g., intramuscular or subcutaneous injection) are inherently inefficient at inducing robust local Trm, primarily eliciting immune responses concentrated in the systemic circulation. This constitutes the immunological bottleneck for most current vaccines being effective at “preventing disease and severe outcomes” but inefficient at “preventing infection and transmission”. This bottleneck has spurred the resurgence and deep investigation of mucosal vaccinology (e.g., intranasal, oral, rectal, or vaginal administration). The core scientific challenges and opportunities coexist: the mucosal environment presents formidable barriers, including physical (mucus layer, tight epithelial junctions), chemical (degradative enzymes, extreme pH), and immunological (a default tolerogenic environment designed to prevent overreaction to commensals and food antigens). Designing vaccines to overcome these obstacles is a major research thrust. For oral vaccines specifically, critical hurdles include surviving the acidic stomach environment, resisting proteolytic enzymes in the gastrointestinal tract, and penetrating the mucus barrier to reach immune-inductive sites like Peyer’s patches. Nanotechnology offers potential solutions, such as encapsulating antigens in polymers resistant to low pH and enzymes, or coating nanoparticles with mucus-penetrating agents (e.g., polyethylene glycol) to facilitate transport through the mucus layer to the underlying epithelium. For intranasal vaccines, while the environment is less harsh, efficient delivery across the nasal epithelium to nasal-associated lymphoid tissue remains a key design parameter. However, even promising mucosal adjuvants (e.g., modified bacterial toxins) have faced setbacks in clinical trials due to safety concerns such as Bell’s palsy (as seen with an intranasal inactivated influenza vaccine adjuvanted with E. coli heat-labile toxin). This highlights the delicate balance between immunogenicity and safety that must be struck for mucosal vaccines to succeed. Examples include using non-toxic subunits of bacterial toxins (e.g., cholera toxin B subunit, mutant forms of E. coli heat-labile toxin) as mucosal adjuvants and targeting molecules [24], or developing mucus-penetrating nanoparticles (e.g., particles coated with mucus-penetrating polymers) [25]. Successful mucosal vaccines can not only provide excellent individual protection but also, by drastically reducing local pathogen replication and shedding, effectively block human-to-human transmission chains, thereby significantly enhancing prevention and control efficacy at the population level. This represents a higher-order goal in infectious disease prevention and a key direction for next-generation vaccine design.

## 3. Integrated Innovative Pathways for Next-Generation Vaccine Design

Deep understanding of immune response network dynamics provides a clear engineering blueprint and tunable parameters for rational vaccine design. Innovation in next-generation vaccines is advancing deeply along three tightly interwoven and interdependent dimensions—antigen, adjuvant, and delivery system—toward high integration, intelligence, and programmability.

### 3.1. Antigen Engineering: From Structural Mimicry to Computationally Guided “Antifragile” Design and Synthetic Immunogen Creation

The antigen, the bullseye and trigger for eliciting specific immune responses, has progressed from early natural extraction or simple recombinant expression into a golden age of rational design based on atomic-level structural information, and is now moving toward a new stage of computational simulation-driven creation. Progress in this field is first exemplified by structural stabilization and conformational locking of natural antigens. For instance, for HIV-1 envelope protein trimers (Env), influenza hemagglutinin (HA), and coronavirus spike (S) proteins, researchers have successfully locked them in the pre-fusion native conformation via point mutations guided by molecular dynamics simulation, introducing non-native disulfide bonds, proline substitutions, or filling hydrophobic cavities [26,27]. This maximizes exposure of key neutralizing epitopes while avoiding induction of non-neutralizing or even harmful antibodies (e.g., antibody-dependent enhancement, ADE).

A more frontier and challenging area is epitope-focused and immune-response steering design, aiming fundamentally to overcome interference from immunodominant regions on the pathogen surface and forcibly focus the immune response onto evolutionarily conserved, functionally critical Achilles’ heels. High-resolution cryo-electron microscopy (Cryo-EM) and X-ray crystallography precisely locate these conserved, often occluded sites (e.g., the conserved stalk region of influenza HA, conserved areas around the CD4 binding site of HIV Env, the lateral side of the coronavirus S protein receptor-binding domain). Subsequent protein engineering enables de-immunodominance design: either physically masking variable immunodominant regions by introducing new glycosylation sites (glycol-engineering); or using a mosaic strategy to computationally integrate conserved sequence fragments from multiple evolutionary clades or subtypes into a single immunogen; or constructing chimeric and grafted antigens by transplanting the target conserved epitope from its native context onto a heterologous, highly stable protein scaffold [28]. Such designs are central to developing universal influenza vaccines and broad-spectrum beta coronavirus vaccines, aiming to induce broadly neutralizing antibodies targeting viral soft spots.

The most revolutionary direction, demonstrating exceptional potential, is multivalent self-assembling nanoparticle vaccines. Genetically fusing genes encoding optimized antigenic domains to carrier proteins that spontaneously assemble into highly symmetrical nanoparticles (e.g., 24-mer ferritin, 60-mer IMX313/Spy Catcher systems, or computationally designed protein scaffolds) enables in vivo expression and self-assembly of “synthetic immunogens” not found in nature [29,30]. These engineered nanoparticles present dozens or even hundreds of antigen copies in precise geometric arrays. Their remarkable multivalency efficiently cross-links BCRs, inducing potent B cell activation signals and significantly lowering the antigen concentration threshold for B cell activation. Their optimized nano-size (20–100 nm) facilitates active transport to lymph nodes via lymphatic vessels and efficient uptake by FDCs and APCs. Their regular, repetitive structures are more readily captured and retained long-term on FDC surfaces via complement- or Fc receptor-mediated mechanisms. In preclinical and early clinical studies targeting influenza, HIV, malaria, RSV, and SARS-CoV-2, such nanoparticle vaccines consistently demonstrate the potential to induce antibody titers, neutralization breadth, and memory durability far surpassing traditional soluble protein vaccines, representing a paradigmatic breakthrough in antigen engineering [31,32,33].

### 3.2. Adjuvant Systems: From Immune Potentiators to Spatiotemporal Response Programmers and Synergistic Network Builders

The evolving role of adjuvants epitomizes modern vaccinology’s shift from “mimicking nature” to “designing and surpassing nature”. New-generation adjuvants are no longer simple immunostimulants but a molecular and signaling toolkit capable of precisely triggering specific innate immune pathways and synergistically programming responses with antigens and delivery systems. Development has moved beyond classic aluminum salts (predominantly Th2-biasing) to a diverse family, including defined TLR agonist families (e.g., TLR4’s MPL, TLR7/8’s Resiquimod, TLR9’s CpG oligos), emerging agonists for intracellular nucleic acid sensors (e.g., cyclic dinucleotides CDNs for cGAS-STING, RNA ligands for RIG-I), and saponin-based adjuvants with unique mechanisms (e.g., QS-21, forming cholesterol-dependent membrane pores and promoting cross-presentation) [34,35]. Despite the promise of novel adjuvants in preclinical models, their translation to clinical use is often hampered by toxicity concerns (e.g., excessive inflammation, pyrogenicity) and manufacturing scalability issues. For instance, many saponin-based adjuvants require complex purification processes and can cause hemolytic side effects, necessitating formulation into immunostimulatory complexes (ISCOMs) to mitigate toxicity. Similarly, synthetic TLR agonists must be carefully dosed to avoid systemic cytokine storms. These challenges underscore the need for iterative optimization and robust safety evaluation in adjuvant development.

However, merely possessing potent signal molecules is insufficient. Rational combination and precise subcellular delivery are key to achieving effective and safe immune programming. Combining adjuvants acting on different pathways or different nodes of the same pathway (e.g., TLR4 agonist MPL combined with saponin QS-21 in the AS01 series, or aluminum salts combined with MPL and CpG in AS04) often yields synergistic effects (1 + 1 > 2), inducing more comprehensive, balanced, and potent mixed Th1/Th2 or cellular immune responses [36,37]. A more sophisticated and increasingly emphasized strategy is using advanced nano-delivery technologies to physically co-encapsulate adjuvant and antigen within the same nanocarrier (e.g., lipid nanoparticles LNPs, polymer nanoparticles, liposomes), ensuring their co-internalization by the same set of APCs in vivo and achieving spatiotemporally synchronized or sequential release in appropriate intracellular compartments (e.g., endosome, lysosome, or cytosol). This co-delivery strategy enables subcellular co-localization of signal and antigen, maximizing the intensity and specificity of synergistic stimulation while minimizing the risk of systemic side effects (e.g., fever, local inflammation) due to adjuvant dissemination. Indeed, the success of mRNA-LNP vaccines is partly attributable to this: LNPs not only protect mRNA, but certain ionizable lipid components, upon protonation in acidic endosomes, promote endosomal escape and can themselves act as adjuvants that activate innate pathways, achieving natural co-delivery and synergy between antigen (mRNA-encoded protein) and adjuvant (LNP components). This exemplifies the deep integration of the three pillars in a licensed product. Another example is the AS01 adjuvant system used in the Shingrix vaccine, where two distinct adjuvants (MPL and QS-21) are co-formulated in liposomes to synergistically enhance both humoral and cellular responses [38].

### 3.3. Delivery Platforms: From Passive Transport Vehicles to Active Immune Modulators and Intelligent Biointerfaces

Delivery systems have radically evolved from their historically passive role of protecting the antigen from degradation and aiding transport to lymphoid organs into multifunctional biointerfaces and modulators capable of actively participating in and precisely regulating the entire immune response process with smart responsive features.

The monumental success of LNPs in mRNA vaccines vividly demonstrates the immense potential and flexibility of this highly customizable, multifunctional platform. LNP performance is not fixed but can be finely tuned through high-throughput screening and rational design of the chemical structures of its four core components: ionizable lipids (determining mRNA encapsulation efficiency, endosomal escape, and adjuvant activity), helper phospholipids (maintaining membrane structure), cholesterol (stabilizing membrane fluidity), and PEGylated lipids (affecting particle stability, circulation time, and immunogenicity) [39]. For example, developing novel biodegradable ionizable lipids (e.g., containing cleavable ester bonds) can improve LNP safety and delivery efficiency; adjusting PEG-lipid chain length, density, and linkage precisely influences PEG shedding kinetics, thereby modulating LNP blood half-life, liver clearance, and targeting to specific cells (e.g., APCs). These designs enable LNPs to be tailor-made for different applications (e.g., prophylactic vaccines, therapeutic cancer vaccines).

However, the use of PEGylated lipids is not without potential drawbacks. While PEGylation enhances particle stability and reduces opsonization, it can also trigger the production of anti-PEG antibodies, which may accelerate the clearance of subsequent doses of PEGylated vaccines (the “accelerated blood clearance” phenomenon) and, in rare cases, contribute to hypersensitivity reactions. This highlights a key consideration in the rational design of delivery systems: the trade-off between pharmacokinetic benefits and potential immunological liabilities. Ongoing research is exploring alternative hydrophilic polymers or biodegradable PEG-lipid conjugates to mitigate these issues.

Viral vectors (e.g., replication-deficient adenoviruses, Modified Vaccinia Ankara MVA, *Vesicular Stomatitis Virus*) constitute an indispensable pole in the vaccine platform arsenal, prized for their high infection efficiency, strong inherent adjuvant activity (carrying abundant PAMPs), and inherent strength in inducing potent and durable T cell immunity. Current research focuses on addressing core clinical bottlenecks: pre-existing immunity (evaded by using rare human adenovirus serotypes, non-human adenoviruses, or mosaic capsid engineering), safety optimization (constructing more replication-deficient vectors by deleting additional viral genes), and achieving cell- or tissue-specific targeting via genetic engineering of capsid/envelope proteins [40,41]. Heterologous prime-boost strategies (e.g., adenovirus vector prime, mRNA, or protein nanoparticle boost) have been extensively validated in preclinical and clinical data to effectively circumvent pre-existing immunity against the vector or interference from neutralizing antibodies generated after the prime. They often elicit stronger, broader (especially against variants) humoral and cellular immune responses than homologous regimens, showcasing the strategic value of platform combinations [42,43].

Furthermore, biomimetic and stimulus-responsive nanocarriers represent another imaginative frontier. For instance, nanoparticles cloaked with membranes from specific leukocytes (e.g., DCs, macrophages) or red blood cells can leverage the source cell’s self-marker and inherent chemotactic properties to evade rapid clearance by the mononuclear phagocyte system and potentially achieve active targeting to inflammatory sites or specific organs. Designing pH-sensitive (dissociating in acidic endosomes/lysosomes), enzyme-sensitive (degrading in enzyme-rich specific tissues), or redox-sensitive (cleaving under high intracellular glutathione concentration) carrier materials can achieve triggered, on-demand release of antigen/nucleic acid in specific tissue microenvironments or intracellular organelles, further enhancing delivery precision, safety, and efficiency [44,45]. These intelligent delivery systems—ranging from tunable LNPs and engineered viral vectors to biomimetic nanocarriers—collectively represent a move toward programmable biointerfaces that actively engage with the immune system, turning the vision of precision vaccines into reality.

## 4. Immunological Strategies and Fundamental Bottlenecks in Addressing Complex Challenges

Despite an increasingly sophisticated and integrated technological toolbox, vaccinology still faces several deep-rooted challenges stemming from the complexity of pathogen-host interactions, the biological constraints of the immune system itself, and public health practicalities. These challenges define the current research frontier and key research priorities.

### 4.1. The Central Role of Adaptive Immunity in Vaccine Protection: Antibodies and T Cells as Complementary Forces

Effective vaccines must elicit both arms of adaptive immunity: humoral (antibody-mediated) and cellular (T cell-mediated) responses, each playing distinct yet complementary roles in protection. Neutralizing antibodies, produced by long-lived plasma cells, provide the first line of defense by blocking pathogen entry into host cells, thereby preventing infection. The quality of this antibody response—its affinity, breadth, and isotype—is shaped by germinal center reactions and is crucial for sterilizing immunity against viruses like SARS-CoV-2 and influenza. However, antibodies alone are often insufficient for controlling intracellular pathogens such as Mycobacterium tuberculosis, HIV, or latent viruses. Here, T cells become indispensable. CD4+ helper T cells, particularly Th1-polarized cells, orchestrate immune responses by secreting cytokines like IFN-γ that activate macrophages and enhance CD8+ T cell function. CD8+ cytotoxic T lymphocytes directly eliminate infected cells by recognizing pathogen-derived peptides presented on MHC class I molecules. Moreover, tissue-resident memory T cells (Trm) stationed at mucosal surfaces provide rapid on-site defense. Therefore, next-generation vaccine design must strategically engage both B cell and T cell compartments. This is achieved through rational antigen design (e.g., inclusion of conserved T cell epitopes), appropriate adjuvant selection (e.g., TLR agonists that promote Th1 polarization), and delivery platforms that enable cross-presentation for CD8+ T cell activation (e.g., viral vectors, certain nanoparticle formulations). The synergy between antibodies and T cells forms the bedrock of durable, broad-spectrum protection.

### 4.2. The Pursuit of Broad-Spectrum Vaccines: Immunological Wisdom and Evolutionary Steering Strategies Against Antigenic Variation

Developing universal vaccines with broad and durable protective efficacy against rapidly evolving viruses (e.g., universal influenza, broad-spectrum coronavirus, HIV vaccines) is a long-standing holy grail. The core immunological dilemma lies in the fact that the virus’s essential functional regions (conserved sites) are often structurally occluded (e.g., protein trimer interfaces, stalk regions) or inherently poorly immunogenic, constituting immunological cold spots; whereas exposed, immunodominant regions are typically hypervariable, and antibodies targeting them are easily escaped by mutation. Overcoming this fundamental dilemma requires multi-layered, sequential combinatorial strategies beyond single antigen design.

First, the aforementioned nanoparticle display, conformational locking, and de-immunodominance design can significantly enhance the immunogenicity and accessibility of conserved epitopes, turning them from “invisible” to “visible” targets. Further, sequential immunization or immune-focusing strategies show promising potential: priming the immune system with one (or a set of) natural or modified antigens (e.g., HA from a circulating strain) establishes an initial B cell response repertoire [46]; subsequently, boosting with computationally designed booster antigens containing a series of evolutionarily relevant variant features or highly focused on conserved core structures aims to guide or shape the evolutionary direction of B cell lineages within GCs. The goal is to steer affinity maturation from recognizing variable, immunodominant epitopes towards recognizing more occluded, conserved core epitopes, ultimately yielding antibodies with ultra-broad neutralizing activity. Additionally, reinforcing cross-reactive T cell immunity is crucial. Designing vaccine components targeting highly conserved internal non-structural proteins (e.g., polymerase PA/PB1, nucleocapsid protein NP) can induce robust and durable CD4+ and CD8+ T cell responses [47]. Unlike antibodies, T cells recognize processed short peptides presented by MHC molecules and are relatively more tolerant to amino acid point mutations. Thus, they provide a robust back-end defense against antigenic drift, playing a key role in controlling disease severity and preventing severe outcomes and death. An ideal broad-spectrum vaccine should simultaneously elicit potent broadly neutralizing antibodies and broad T cell immunity, forming a multi-layered protective network.

### 4.3. Constructing Durable Immunity: Understanding and Regulating Memory Maintenance, Niches, and Refreshment Mechanisms

The relatively rapid waning of neutralizing antibody titers from COVID-19 vaccines within months and the annual update requirement for influenza vaccines point to the universal and critical challenge of immune memory durability. Memory persistence is not determined by a single factor but is a dynamic process influenced by multiple intertwined elements, including: the strength and quality of the primary immunization (especially the sufficiency and duration of the GC reaction); the nature of the antigen itself (e.g., stability, whether it is a live vaccine); the capacity and supporting signals of the specialized microenvironment (niche) where memory cells (especially long-lived plasma cells) reside in tissues like bone marrow; and the inevitable age-related immunosenescence (involving declines in hematopoietic stem cell function, impaired lymphopoiesis, APC function, etc.).

To achieve long-lasting or even lifelong protection, frontier research not only focuses on optimizing primary immunization to generate more high-quality memory precursor cells but also explores new paradigms for maintaining and refreshing established memory. Heterologous boosting (using different technology platforms for boost) has proven effective in reactivating GC reactions, driving further evolution, expansion, and continued affinity maturation of the memory B cell pool, while potentially inducing new dominant clones [48]. A more innovative concept involves developing long-acting sustained-release formulations or self-boosting vaccines. For example, using biodegradable polyester microspheres or polysaccharide/polypeptide-based hydrogels loaded with antigen/adjuvant, injected subcutaneously or intramuscularly to form an antigen depot, enabling sustained or pulsatile low-dose antigen release over weeks to months [49,50]. This strategy aims to mimic the physiological state of chronic infection or repeated low-level natural exposure, potentially gently and persistently stimulating memory B and T cells to maintain their functional state and numbers, preventing them from entering deep quiescence or undergoing apoptosis due to prolonged antigen absence. This offers a theoretical possibility and application prospects for vaccines with dosing intervals of several years or more.

### 4.4. The Challenge of Complex Pathogens: Activating Multi-Dimensional, Tissue-Specific, and Durable Cellular Immunity

For complex intracellular pathogens like *Mycobacterium tuberculosis*, *Plasmodium* parasites, *Leishmania*, and retroviruses like HIV that integrate into the host genome and establish latent reservoirs, vaccine development challenges increase exponentially. These pathogens have complex life cycles, multiple active immune evasion mechanisms (e.g., antigenic variation, interference with antigen presentation, suppression of immune cell function), and long-term latent survival capabilities. Against them, ideal vaccines often need to elicit potent, multifunctional cellular immunity capable of effectively homing to infection-specific tissues, particularly Th1-type CD4+ T cells (providing crucial help and secreting effectors like IFN-γ) and cytotoxic CD8+ T cells (directly killing infected cells).

This imposes exceptionally stringent and specific demands on vaccine platforms: First, high-efficiency cytosolic antigen delivery must be achieved to promote cross-presentation primarily via the MHC class I pathway, effectively activating CD8+ T cells [51,52]. Viral vectors (e.g., adenovirus, MVA) or certain optimized LNP/mRNA combinations with efficient endosomal escape have inherent or engineered advantages here. Second, the adjuvant used must strongly induce key cytokines like IL-12, type I IFNs, and IL-15, which drive Th1 differentiation and CD8+ T cell expansion, survival, and tissue-resident memory formation [53]. Furthermore, enabling vaccine-induced effector and memory T cells to effectively home and long-term reside at primary infection sites (e.g., lung interstitium for TB, liver for malaria) is a complex biological consideration in design, potentially involving incorporating specific chemokines or using mucosal vaccination routes. These stringent and multifaceted requirements make effective vaccine development against such pathogens exceptionally difficult and protracted. However, they also serve as an impetus, continuously driving immunology theory (e.g., tissue-specific immunity, balance of exhaustion and memory) and vaccine platform technology (e.g., novel vectors, potent cellular immunity adjuvants) to deeper and broader levels.

## 5. Towards the Future: Systems Vaccinology, Artificial Intelligence, and Personalized Vision

The future of vaccinology is witnessing a profound convergence with the era of data science, computational power, and precision medicine, pointing toward a new direction of greater predictability, efficiency, intelligence, and personalization. This is not merely a technological overlay but a holistic upgrade of the research and development paradigm.

### 5.1. Systems Vaccinology: Decoding the Immune Response Black Box from a Holistic Perspective, Achieving the Leap from Correlation to Causation

As an emerging interdisciplinary field, systems vaccinology employs high-throughput omics technologies (transcriptomics, proteomics, metabolomics, epigenomics, single-cell immune repertoire sequencing, etc.) to provide global, unbiased, dynamic quantitative descriptions of immune states at different time points before and after vaccination, aiming to generate vast amounts of multi-layered data [54]. Its grand goal is twofold: first, to discover translatable biomarkers capable of early and accurate prediction of vaccine protective efficacy (e.g., specific antibody subclasses or glycosylation patterns, characteristic gene expression signatures, frequency and phenotype of key immune cell subsets) to accelerate clinical trial evaluation and potentially surrogate some traditional endpoints; second, and more fundamentally, to use these big data to construct biologically informed computational and network models, revealing the intrinsic regulatory logic, key nodes, and molecular basis of individual differences in immune responses from innate activation to memory establishment. For example, systematically comparing multi-omics profiles of young vs. elderly, high vs. low vaccine responders, or recipients of different adjuvant regimens can identify key immune pathway defects, cellular metabolic states, or regulatory network perturbations affecting vaccine performance [55]. These insights can provide direct molecular targets and design rationales for reverse-engineering optimized vaccine formulations (e.g., adjusting adjuvant type/dose, adding specific immunostimulatory signals) for specific vulnerable populations (e.g., the elderly, immunocompromised), moving vaccine design from trial-and-error toward mechanism-based prediction.

### 5.2. AI-Driven: Reshaping the Vaccine R&D Paradigm, Speed, and Innovation Boundaries

Artificial intelligence and machine learning are permeating and reshaping almost every stage of vaccine R&D from concept to product, becoming an indispensable core accelerator and innovation engine. For instance, deep learning-based protein structure prediction (e.g., AlphaFold2) enables rapid design of stabilized antigens, while generative models can explore vast chemical spaces to optimize lipid nanoparticles for mRNA delivery. In the antigen design phase, revolutionary breakthroughs in deep learning-based protein structure prediction algorithms (e.g., AlphaFold2, RoseTTAFold) enable rapid, accurate prediction of antigen and antigen–antibody complex 3D structures, thereby significantly accelerating the process of rational antigen design [56]. AI can also be used for de novo design of novel protein scaffolds not found in nature, with ultra-high stability and specific antigen display capabilities [57]. In the mRNA vaccine field, machine learning models can optimize coding sequences for codon usage, 5′ and 3′ untranslated region (UTR) sequences, and poly A tail length to maximize translation efficiency, mRNA stability, and final protein expression. In adjuvant and delivery system development, AI (particularly deep learning and generative models) can learn and predict the complex nonlinear relationships between specific lipid molecular structures, polymer compositions, and in vivo immunological outcomes (e.g., antibody titers, Th1/Th2 balance, cellular immunity strength, safety metrics) from vast chemical libraries, in vitro high-throughput screening data, and in vivo experimental data, thereby efficiently searching, designing, and optimizing new molecules and formulations with superior performance in virtual space [58]. AI transforms the traditionally time-consuming, costly “trial-and-error” intensive R&D into a fast-iterating, intelligent cycle of predict-design-synthesize-validate-learn, drastically shortening development timelines, reducing failure costs, and potentially exploring novel design spaces beyond human intuition.

### 5.3. Personalization and Platformization: Dual Pillars and a Convergent Ecosystem for Future Threats

The combination of individual variation insights derived from systems biology and rapid design capabilities from AI naturally leads to the vision of personalized vaccines. While tailoring completely different vaccines for each healthy individual in preventive infectious disease contexts faces immense cost and logistical challenges for mass application, stratified or population-personalized strategies are showing scientific necessity and preliminary technical feasibility in specific high-value or high-risk scenarios. Examples include customizing booster vaccine formulations with more potent adjuvants, specific immunostimulatory signal combinations, or higher antigen doses for immunocompromised individuals (e.g., transplant recipients, people living with HIV, chemotherapy patients) or the aging elderly [59]; or optimizing the combination of T cell epitopes included in a vaccine based on the distribution of major Human Leukocyte Antigen alleles in a specific geographic region or population to ensure effective T cell responses covering the vast majority of individuals in the target group.

However, the path to widespread personalized vaccination is fraught with barriers. Logistically, manufacturing individualized doses on demand would require a paradigm shift from centralized batch production to distributed, perhaps even point-of-care, manufacturing. Regulatory frameworks are currently designed for fixed products; approving dynamically personalized vaccines would necessitate new paradigms for quality control and clinical validation. Economically, the high cost of personalized biologics could exacerbate global health inequities, limiting access in low-resource settings. Therefore, while the scientific vision is compelling, its realization will depend on parallel innovations in manufacturing, regulation, and financing to ensure that precision does not come at the expense of equity.

Parallel and complementary to the precision approach of personalization is the continuous optimization of platform technologies. Modular mRNA-LNP platforms, rapid cloning and expression systems (e.g., baculovirus-insect cell, plant transient expression), and universal self-assembling nanoparticle scaffolds together form the cornerstone for rapid response and large-scale production against emerging variants or even completely unknown “Disease X” pathogens [60,61]. Platformization ensures speed and scale in vaccine R&D and manufacturing, while personalization (or stratification) ensures precision and effectiveness in the complex real world. In the future, we are likely to witness the formation of a hybrid ecosystem: core platform technologies address broad, sudden public health threats for rapid deployment, while precision-optimized solutions based on population or individual characteristics are used to address specific high-risk scenarios, enhance protection in key populations, or serve as preferred booster strategies. This “platform-precision” dual-drive model will significantly enhance society’s overall resilience and efficacy against infectious disease threats.

## 6. Conclusions

We stand at the threshold of a new era in vaccinology, forged by foundational insights into innate immune programming and germinal center dynamics, and propelled by convergent advances in structural biology, materials science, and computational biology. The emerging blueprint envisions vaccines not as simple biological products but as “synthetic immunotherapeutic agents”—rooted in a quantitative understanding of the immune system and created through integrated, programmable bioengineering to deliver precision protection. The three pillars of next-generation design—computationally engineered immunogens, spatiotemporally controlled adjuvant systems, and intelligent delivery platforms—must operate in synergy to overcome the limitations of traditional empiricism. Critical challenges remain: we must decipher the rules governing immune memory durability to extend protection beyond months, develop effective mucosal vaccines that block transmission at the portal of entry, and harness systems vaccinology and AI to predict and personalize immune responses. Moreover, translating these technological advances into globally accessible interventions requires sustained commitment to equity and infrastructure. The COVID-19 pandemic served as an unprecedented stress test and catalyst, proving that convergent science can rapidly reshape our defenses. Looking ahead, continued exploration of fundamental immunology, coupled with deep integration across disciplines, holds the potential to finally control persistent threats like HIV, tuberculosis, and malaria, and to prepare us for future pathogens. Ultimately, this endeavor transcends the laboratory—it represents a profound collective effort to employ our wisdom and ethics in building a healthier, more resilient future for all.

## Data Availability

No new data were created or analyzed in this study. Data sharing is not applicable to this article.
